# Retraction Note: Expression of Functional Anti-p24 scFv 183-H12-5C in HEK293T and Jurkat T Cells

**DOI:** 10.15171/apb.2018.082

**Published:** 2018-11-29

**Authors:** Mohammad Tasyriq Che Omar

**Affiliations:** ^1^Cluster of Oncology and Radiological Sciences, Advanced Medical and Dental Institute, Universiti Sains Malaysia, 13200 Kepala Batas, Pulau Pinang, Malaysia.; ^2^Biology Program, School of Distance Education, Universiti Sains Malaysia, 11800 USM, Pulau Pinang, Malaysia.

The main and corresponding author, “Mohammad Tasyriq Che Omar” submitted this article to the “Advanced Pharmaceutical Bulletin” Journal on 20 of April 2017 and it was published after positive reviews in: Adv Pharm Bull, 2017, 7(2), 299-312, doi: 10.15171/apb.2017.036.

Recently we were informed by an email that this work was carried out under direct supervision of a researcher in the “Advanced Medical and Dental Institute, Universiti Sains Malaysia” between 2013-2016. On the submitted and published manuscript “Mohammad Tasyriq Che Omar” did not include the name of his supervisor as an author, who substantially contributed to conception and design of the work. No permission was obtained from the supervisor for submission of this work.

We contacted “Mohammad Tasyriq Che Omar” regarding this issue and he admitted this mistake. He requested to add his supervisor’s name on the published paper. This could not be happened as it is against the current regulations and professional ethics.

Publication of this work without the knowledge, consent, and permission of the PhD supervisor and without his name is unacceptable and reflective of extremely poor professional ethics. The editors of the “Advanced Pharmaceutical Bulletin” Journal who act according to the COPE Code of Conduct, consider this an infringement of professional ethics and the decision has been made to retract the article published in the Journal.

The editors of “Advanced Pharmaceutical Bulletin” Journal are sorry for any inconveniences caused to the reviewers, editorial staff and to the readers.

Prof Hadi Valizadeh

“Advanced Pharmaceutical Bulletin” Editor-in-Chief

